# Regulation of murine skeletal muscle growth by STAT5B is age- and sex-specific

**DOI:** 10.1186/s13395-019-0204-3

**Published:** 2019-06-24

**Authors:** Ryan G. Paul, Alex S. Hennebry, Marianne S. Elston, John V. Conaglen, Chris D. McMahon

**Affiliations:** 1 AgResearch Ltd, Ruakura Research Centre, Private Bag 3123, Hamilton, New Zealand; 2 Faculty of Medical & Health Sciences, Waikato Clinical Campus, University of Auckland, Private Bag 3200, Hamilton, 3240 New Zealand

**Keywords:** STAT5B, Sexually dimorphic growth, GH, IGF1, Myostatin, AR, ERα, SOCS2, CIS

## Abstract

**Background:**

Sexually dimorphic growth has been attributed to the growth hormone (GH)/insulin-like growth factor 1 (IGF1) axis, particularly GH-induced activation of the intracellular signal transducer and activator of transcription 5B (STAT5B), because deletion of STAT5B reduces body mass and the mass of skeletal muscles in male mice to that in female mice. However, it remains unclear why these effects are sex- and species-specific, because the loss of STAT5B retards growth in girls, but not in male mice. Our objectives were to determine whether sexually dimorphic growth of skeletal muscle persisted in STAT5B^−/−^ mice and investigate the mechanisms by which STAT5B regulates sexually dimorphic growth.

**Methods:**

Blood and skeletal muscle were harvested from male and female STAT5B^−/−^ mice and their wild-type littermates from the onset of puberty to adulthood.

**Results:**

Growth of the skeleton and skeletal muscles was retarded in both sexes of STAT5B^−/−^ mice, but more so in males. Although reduced, sexually dimorphic growth of skeletal muscle persisted in STAT5B^−/−^ mice with an oxidative shift in the composition of myofibres in both sexes. Concentrations of IGF1 in blood and skeletal muscle were reduced in male STAT5B^−/−^ mice at all ages, but only in female STAT5B^−/−^ mice at the onset of puberty. Expression of androgen receptor (AR) and oestrogen receptor alpha (ERα) mRNA and protein was reduced in skeletal muscles of male and female STAT5B^−/−^ mice, respectively. Loss of STAT5B abolished the sexually dimorphic expression of myostatin protein and *Igf1*, *Ar*, *Erα*, *suppressor of cytokine signalling 2* (*Socs2*), and cytokine-inducible SH2-containing protein (*Cis*) mRNA in skeletal muscle.

**Conclusions:**

STAT5B appears to mediate GH signalling in skeletal muscles of male mice at all ages, but only until puberty in female mice. STAT5B also appears to mediate the actions of androgens and oestrogens in both male and female mice, but sexually dimorphic growth persists in STAT5B^−/−^ mice.

**Electronic supplementary material:**

The online version of this article (10.1186/s13395-019-0204-3) contains supplementary material, which is available to authorized users.

## Introduction

Sexually dimorphic growth of skeletal muscle is evident in most mammals from puberty, with males developing a larger body size and muscle mass, with more fast-twitch and less slow-twitch myofibres than females [1, 2]. These differences have been attributed to the actions of the growth hormone (GH)/insulin-like growth factor 1 (IGF1) axis, the major regulator of post-natal growth, because deletion of either the GH receptor (GHR) or IGF1 in mice abolishes the sexual dimorphism of body size [3, 4]. The premise is that GH binds to the GHR, activating the signal transducer and activator of transcription (STAT) family members STAT1, − 3, 5A, and 5B, which form homodimers and heterodimers that regulate the transcription of IGF1 and other target genes [5]. The actions of GH on skeletal muscle appear to be predominantly mediated by STAT5A and STAT5B, because local deletion of STAT5A and STAT5B reduces post-natal muscle growth, while global deletion results in a similar phenotype to the GHR knockout mouse [4, 6]. Of the two, STAT5B appears to be the principle transcription factor regulating sexually dimorphic growth, because removal of STAT5B reduces body mass and the absolute mass of skeletal muscles in male mice to that in female mice [7–9]. The abolition of sexually dimorphic growth in STAT5B^−/−^ mice has been attributed to reduced circulating concentrations of IGF1 and increased concentrations of myostatin in skeletal muscles of only male mice [7–9]. IGF1 and myostatin are the key regulators of skeletal muscle growth, and both are directly regulated by GH via STAT5B [9]. While IGF1 is the major anabolic post-natal growth factor, myostatin restricts the development of skeletal muscle and promotes the development of adipose tissue by inhibiting the signalling of IGF1 [10–12].

However, attributing the sexually dimorphic growth of skeletal muscle to the regulation of IGF1 and myostatin by STAT5B creates important paradoxes. In contrast to mice, it is not clear why inactivating mutations in the *Stat5b* gene in humans reduce growth and circulating concentrations of IGF1 in both sexes [13]. Similarly, unlike the absence of STAT5B alone, it is unclear why sexually dimorphic growth persists in mice with deletion of both STAT5A and STAT5B in skeletal muscle (STAT5M^−/−^), when STAT5A is considered to have no role in growth [4, 6]. Furthermore, circulating IGF1 is likely not required for normal growth, and sexually dimorphic growth of mice persists in mice with overexpression of IGF1 and/or absence of myostatin [14, 15]. Importantly, previous reports on the role of STAT5B in murine skeletal muscle growth have not accounted for the reduced skeletal size and increased adiposity of GH deficiency, or extended beyond 12 weeks of age into adulthood [7–9]. Therefore, either sexually dimorphic growth of skeletal muscle persists in STAT5B^−/−^ mice when changes in body composition are accounted for, or STAT5B regulates sexually dimorphic growth by mechanisms other than altering IGF1 and myostatin activity.

Sexually dimorphic growth of skeletal muscle has been attributed to opposing actions of the gonadal steroids, with androgens promoting and oestrogens inhibiting growth [16, 17]. Accordingly, deletion of either the androgen receptor (AR) or the oestrogen receptor alpha (ERα) reduces the sexual dimorphism of skeletal muscle [18, 19]. GH regulates the transcription of the *Ar* and *Erα* genes in skeletal muscle and other tissues [20, 21], but it is not known whether the expression of these receptors is reduced and, thereby, whether the actions of the gonadal steroids are decreased in STAT5B^−/−^ mice. Sexually dimorphic growth has also been shown to be due to differences in the inhibition of GH signalling between sexes by suppressor of cytokine signalling 2 (SOCS2) and cytokine-inducible SH2-containing protein (CIS) [22, 23]. However, it is not known whether the expression of SOCS2 and CIS in skeletal muscle is sexually dimorphic or whether the expression is regulated by STAT5B. Consequently, the aims of our study were twofold: (1) to determine whether sexually dimorphic growth of skeletal muscle persists in STAT5B^−/−^ mice when adjusting for changes in skeletal size and (2) to determine whether STAT5B regulates the expression of IGF1, AR, ERα, SOCS2, and CIS in skeletal muscle.

## Materials and methods

### Animals

Male and female STAT5B^−/−^ mice (C57BL/6 strain) and wild-type littermates were sacrificed at three time points: the onset of puberty (6 weeks of age), the end of puberty (12 weeks of age), and in early adulthood (24 weeks of age) by CO_2_ asphyxiation and cervical dislocation (*n* = 8 for each sex and genotype at each time point). Mice were weighed, and blood was collected by cardiac puncture. The hindlimb biceps femoris (BF), quadriceps, gastrocnemius, tibialis anterior (TA), extensor digitorium longus (EDL), and soleus muscles were excised, weighed, snap-frozen in liquid nitrogen and stored at − 80 °C. The soleus is a slow-twitch muscle, the TA and EDL are fast-twitch muscles, and the quadriceps, BF, and gastrocnemius muscles have a mixed composition of myofibres [24]. Nasoanal (size of axial skeleton), tibia, and femur (appendicular skeleton) lengths were measured with digital callipers, and the masses of the gonadal and inguinal fat pads were recorded. The mean mass of each muscle group was normalised to the bone length upon which the muscle acted, to allow a comparison for the specific effects on the growth of skeletal muscle [25, 26]. The mass of both fat pads was normalised to total body mass, to allow a calculation of visceral (peri-gonadal) and subcutaneous (inguinal) fat. Plasma was harvested and stored at − 20 °C. All mice were maintained under a photoperiod of 14 h light to 10 h dark and had standard mouse chow (Specialty Feeds, Glen Forrest, Australia) and water ad libitum*.* There were no differences in age or litter size between groups at each time point (Additional file 1: Table S1).

### RNA extraction and real-time PCR

Total RNA was isolated from frozen whole quadriceps muscles using the TRIzol® protocol as previously described [9]. Concentrations and purity of RNA were determined by UV absorbance at 260/280 nm using a Nanodrop® 1000 spectrophotometer. Integrity of RNA was determined by running 1 μg of isolated RNA on an agarose gel with visualisation under UV light (GelDoc). Total RNA (2 μg) from each sample was reverse transcribed (RT) using oligo (dt) primers and SuperScript® III reverse transcriptase (Life Technologies, Carlsbad, California, USA) as per the manufacturer’s instructions. RT reactions were diluted 10-fold, and real-time PCR was performed using a Roche LightCycler® 2.0 as previously described [9]. The sequences of primers used and size of the amplicons are listed in Additional file 2: Table S2. Concentrations of target cDNA were normalised to concentrations of total ssDNA for each RT sample using a Quant-iT™ Oligreen® ssDNA kit (Life Technologies) as per the manufacturer’s instructions [27].

### Protein extraction and Western blot analysis

Protein was extracted from the quadriceps muscles, and Western blotting was performed as previously described [9] using antibodies listed in Additional file 3: Table S3. The relative abundance of the target protein for each sample was normalised to the abundance of total protein, as determined by densitometric analysis of multiple bands in high-resolution scanned images of the Ponceau stain [28].

### MHC protein electrophoresis

Crude lysate samples from the quadriceps muscles were diluted in an 8-M urea/2-M thiourea buffer for MHC protein electrophoresis as previously described [29].

### Plasma and skeletal muscle IGF1 assay

Concentrations of IGF1 protein in plasma and homogenates of quadriceps muscle were determined using a mouse/rat IGF1 Quantikine ELISA (R&D Systems, Minneapolis, USA) as per the manufacturer’s instructions [30]. Concentrations of IGF1 in muscle were normalised to the total protein concentration in each homogenate.

### Statistical analysis

Data were analysed by general ANOVA using GenStat v16 software (VSN International Ltd) with genotype, sex, and age as treatment terms. Residual plots were used to determine whether log transformation was required to stabilise the variance. Post hoc analyses were performed using Fisher’s unprotected test of least significant difference (LSD), which was restricted to intentional comparisons between groups. Two-tailed Student’s *t* tests were used for direct comparisons when there were only 2 variables. Significance was determined as a *P* value < 0.05, and data is presented as mean ± SEM.

## Results

### STAT5B regulates the growth of both male and female mice

**Fig. 1:**
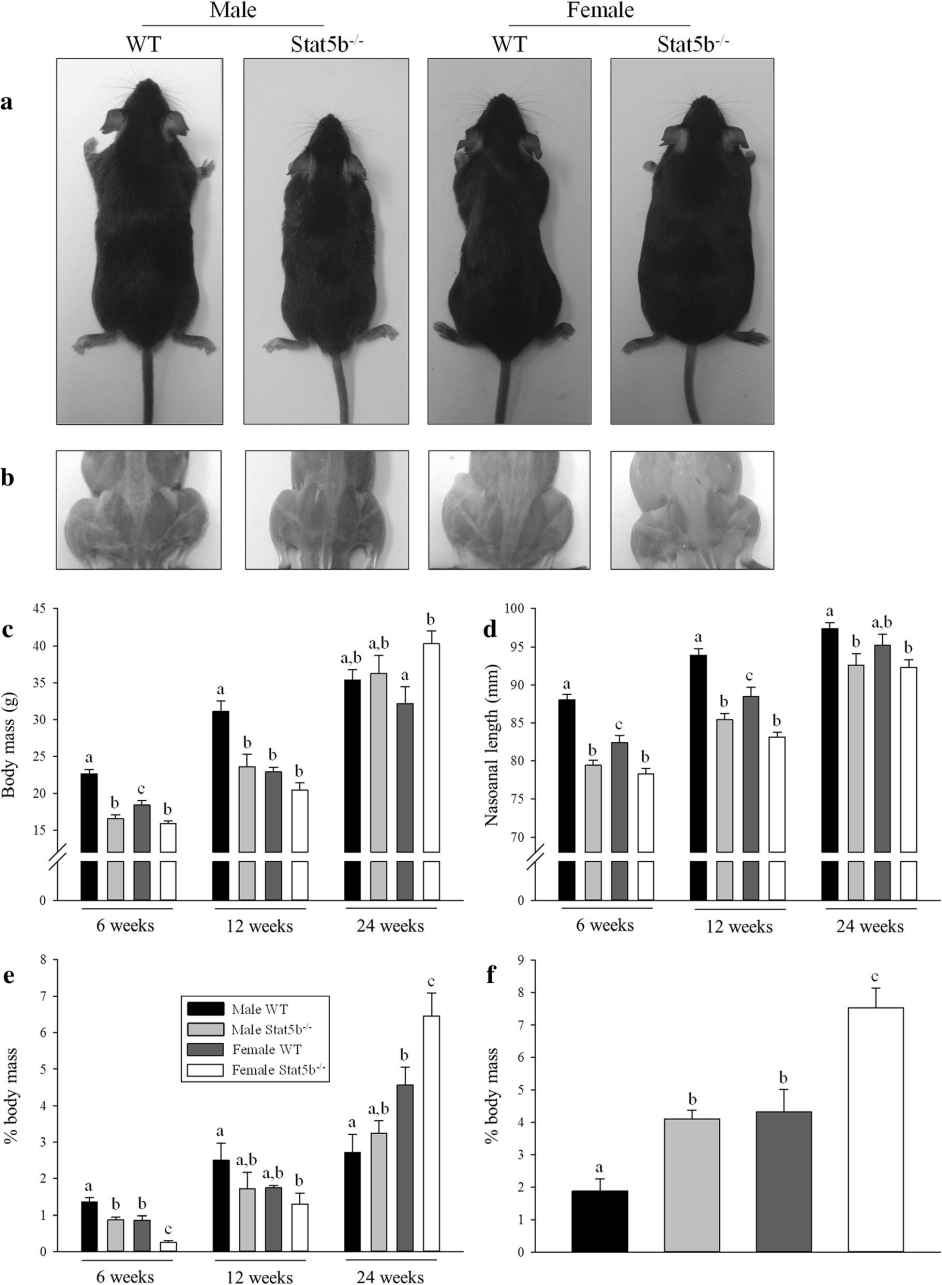
Differences in the skeletal size and body composition between wild-type (WT) and STAT5B^−/−^ mice. Photos of male and female wild-type and STAT5B^−/−^ littermates at 24 weeks of age demonstrate the differences in **a** body size and **b** musculature of the hindlimbs. Mean (± SEM) values of **c** body mass, **d** nasoanal length, and **e** perigonadal fat pad as a percentage of body mass in male WT (black bars), male STAT5B^−/−^ (light grey bars), female WT (dark grey bars), and female STAT5B^−/−^ (white bars) mice at 6, 12, and 24 weeks of age (*n* = 8 per group). **f** Mean (± SEM) percentage of the inguinal fat pad of body mass of WT and STAT5B^−/−^ mice at 24 weeks of age. Unlike letters within each graph denote significant differences (*P* < 0.05) between groups at each age only

The body mass, the size of the axial and appendicular skeletons, the absolute mass of skeletal muscles, and the percentage of visceral fat were reduced in both sexes of STAT5B^−/−^ mice at 6 weeks of age (*P* < 0.01 versus wild-type littermates; Fig. 1c–e and Table 1). The growth retardation of the axial and appendicular skeleton and all hindlimb muscles in male STAT5B^−/−^ mice persisted at all time points (*P* < 0.05 versus wild-type littermates; Table 1). In contrast, only the growth of the appendicular skeleton and the TA and EDL muscles was reduced in female STAT5B^−/−^ mice at 24 weeks of age (*P* < 0.01 versus wild-type littermates; Table 1). The body mass of adult STAT5B^−/−^ mice at 24 weeks of age was not smaller than their wild-type littermates due to increased subcutaneous adiposity in males and both increased visceral and subcutaneous adiposity of females (*P* < 0.05; Fig. 1c, e, f).

**Table 1:** Lengths of the hindlimb bones (mm) and absolute mass of the hindlimb muscles (mg) of wild-type (WT) and STAT5B^−/−^ mice

Age	Sex	Genotype	Tibia	Femur	BF	Gast	TA	EDL	Sol	Quad
6 weeks	M	WT	16.8 ± 0.2^a^	13.1 ± 0.3^a^	118 ± 4^a^	107 ± 2^a^	39 ± 1^a^	8.3 ± 0.2^a^	6.6 ± 0.3^a^	163 ± 4^a^
		STAT5B^−/−^	15.8 ± 0.2^b^	12.0 ± 0.2^b^	75 ± 3^b^	73 ± 2^bc^	27 ± 1^b^	6.2 ± 0.5^b^	5.6 ± 0.7^abc^	106 ± 2^b^
	F	WT	16.4 ± 0.2^a^	12.7 ± 0.3^a^	84 ± 4^c^	78 ± 3^b^	31 ± 1^c^	6.3 ± 0.3^b^	5.0 ± 0.2^b^	119 ± 6^c^
		STAT5B^−/−^	15.9 ± 0.1^b^	12.2 ± 0.1^b^	71 ± 3^b^	68 ± 2^c^	26 ± 1^b^	6.0 ± 0.4^b^	4.4 ± 0.1^c^	97 ± 2^d^
12 weeks	M	WT	18.2 ± 0.2^a^	14.9 ± 0.1^a^	162 ± 8^a^	139 ± 2^a^	49 ± 1^a^	10.8 ± 0.3^a^	8.0 ± 0.5^a^	234 ± 5^a^
		STAT5B^−/−^	16.6 ± 0.2^b^	13.5 ± 0.2^b^	110 ± 4^b^	95 ± 3^bc^	35 ± 1^b^	7.4 ± 0.3^b^	6.0 ± 0.3^b^	143 ± 4^b^
	F	WT	17.7 ± 0.2^c^	14.5 ± 0.1^c^	121 ± 2^c^	101 ± 4^b^	39 ± 2^c^	10.1 ± 0.6^a^	6.9 ± 0.5^ab^	163 ± 4^c^
		STAT5B^−/−^	16.7 ± 0.2^b^	13.6 ± 0.1^b^	107 ± 4^d^	89 ± 2^c^	32 ± 1^d^	7.7 ± 0.2^b^	6.4 ± 0.5^b^	135 ± 2^b^
24 weeks	M	WT	18.1 ± 0.1^a^	15.0 ± 0.1^a^	179 ± 4^a^	148 ± 3^a^	57 ± 2^a^	12.3 ± 0.8^a^	9.7 ± 0.5^a^	245 ± 7^a^
		STAT5B^−/−^	17.2 ± 0.2^b^	14.1 ± 0.1^b^	131 ± 7^b^	115 ± 4^b^	42 ± 1^b^	8.9 ± 0.3^b^	8.0 ± 0.3^b^	173 ± 6^b^
	F	WT	18.4 ± 0.2^a^	15.3 ± 0.2^a^	138 ± 9^b^	117 ± 5^b^	45 ± 2^b^	9.6 ± 0.4^b^	7.8 ± 0.3^bc^	187 ± 6^b^
		STAT5B^−/−^	17.4 ± 0.1^b^	14.4 ± 0.2^b^	111 ± 9^c^	111 ± 3^b^	36 ± 2^c^	7.5 ± 0.3^c^	7.1 ± 0.2^c^	168 ± 6^c^

Values are presented as mean ± SEM. *Abbreviations*: *BF* biceps femoris, *Gast* gastrocnemius, *TA* tibialis anterior, *EDL* extensor digitorium longus, *Sol* soleus, *Quad* quadriceps. Unlike letters within each column denote significant differences (*P* < 0.05) between groups at each age only

### Sexual dimorphism of skeletal muscle is reduced but persists in STAT5B^−/−^ mice

**Fig. 2:**
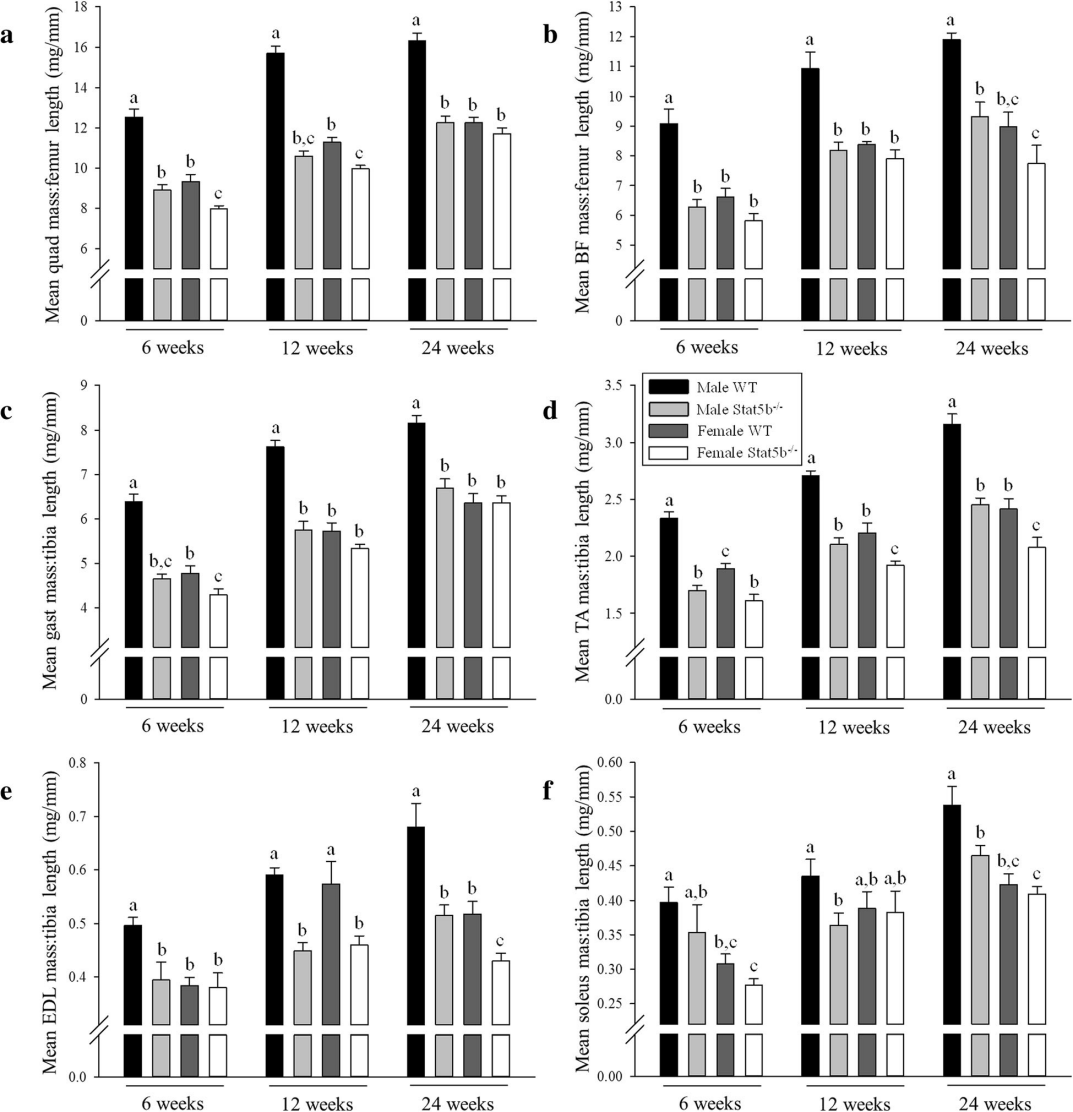
Normalised mass of hindlimb muscles in wild-type (WT) and STAT5B^−/−^ mice. Mean (± SEM) mass of **a** quadriceps, **b** biceps femoris, **c** gastrocnemius, **d** tibialis anterior, **e** extensor digitorium longus, and **f** soleus in male WT (black bars), male STAT5B^−/−^ (light grey bars), female WT (dark grey bars), and female STAT5B^−/−^ (white bars) mice at 6, 12, and 24 weeks of age (*n* = 8 per group). Unlike letters within each graph denote significant differences (*P* < 0.05) between groups at each age only

**Fig. 3:**
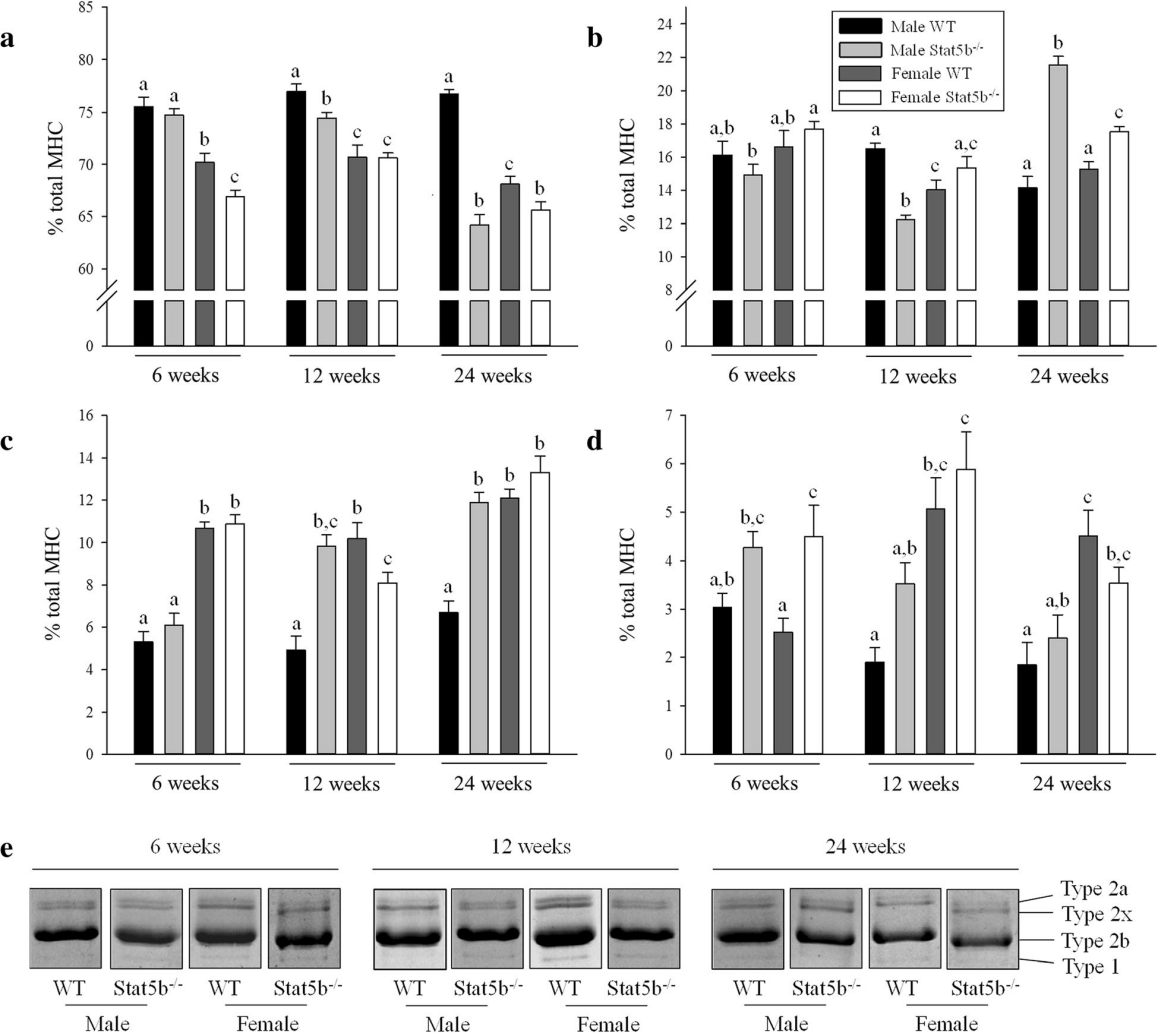
Differences in the composition of myofibres in quadriceps muscles of wild-type (WT) and STAT5B^−/−^ mice. Mean percentages (± SEM) of total myosin heavy chain (MHC) fibres for **a** type 2b, **b** type 2x, **c** type 2a, and **d** type 1 myofibres in quadriceps muscles of male WT (black bars), male STAT5B^−/−^ (light grey bars), female WT (dark grey bars), and female STAT5B^−/−^ (white bars) mice at 6, 12, and 24 weeks of age (*n* = 8 per group). Unlike letters within each graph denote significant differences (*P* < 0.05) between groups at each age only. **e** Representative bands of the MHC gel electrophoresis for each group are also presented

Sexually dimorphic growth of all hindlimb muscles was evident in wild-type mice at all time points (Fig. 2a–f). In STAT5B^−/−^ mice, sexually dimorphic growth was abolished in the quadriceps and gastrocnemius muscles (Fig. 2a, c), but although reduced, it persisted in the BF, TA, EDL, and soleus muscles until 24 weeks of age (*P* < 0.05; Fig. 2b, d–f). Sexual dimorphism in the composition of myofibres was also evident in quadriceps muscles of wild-type mice with a greater proportion of fast-twitch type 2b myofibres in males and an increased proportion of slow-twitch type 1 and 2a myofibres in females (*P* < 0.05; Fig. 3a, c, d). There was an oxidative shift in the composition of myofibres in STAT5B^−/−^ mice with a decreased proportion of type 2b myofibres and an increased proportion of type 2x (both sexes) and 2a (males only) myofibres (*P* < 0.05 versus wild-type littermates; Fig. 3a–c). As a result, the sexually dimorphic composition of myofibres in STAT5B^−/−^ mice was evident for type 2x myofibres, but was abolished for type 2b, 2a, and 1 myofibres in adult mice at 24 weeks of age (Fig. 3a–d).

### STAT5B regulates concentrations of IGF1 in both sexes before puberty

**Fig. 4:**
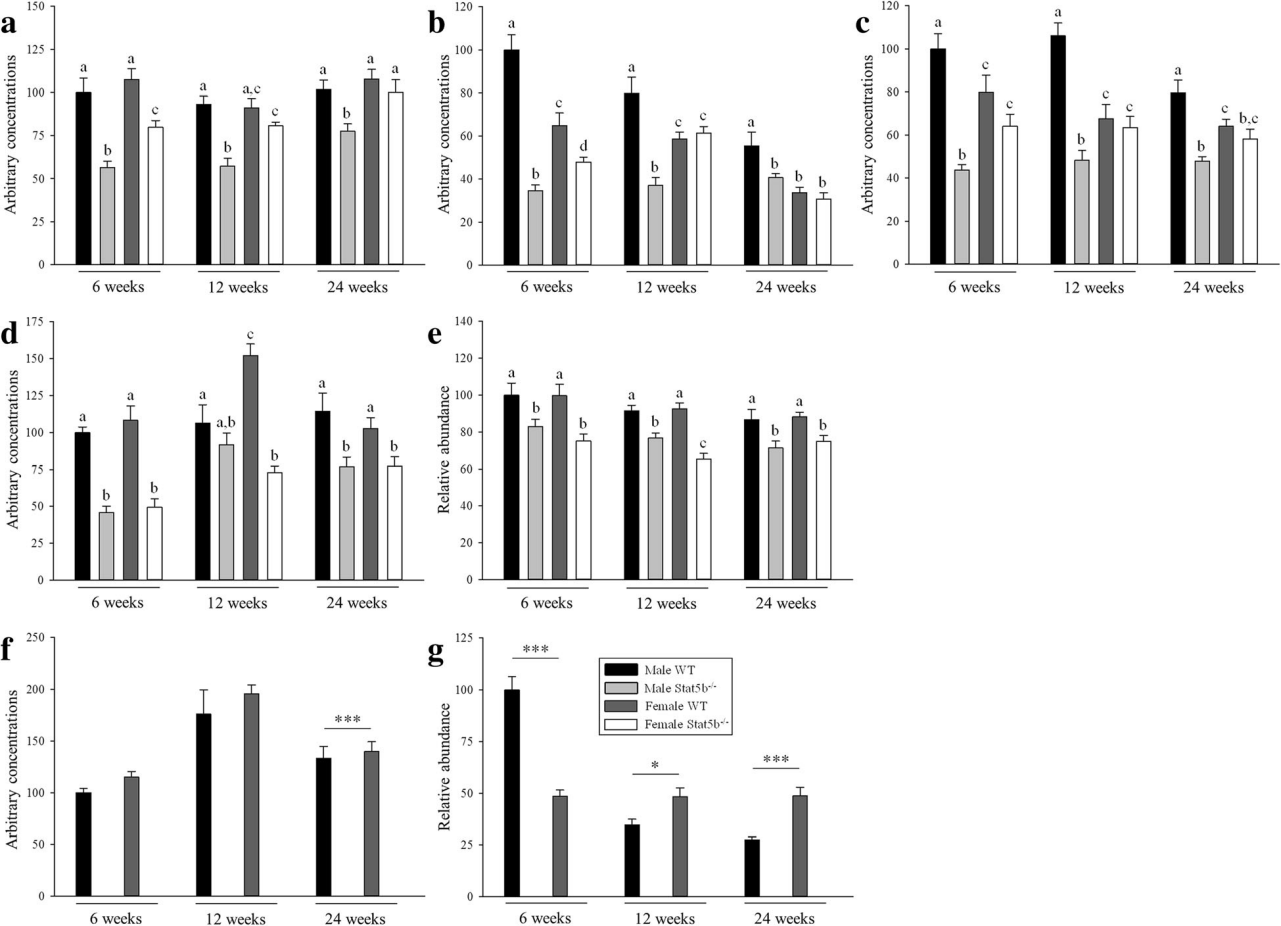
Differences in plasma levels of IGF1 and the expression of IGF1, STAT5A, and STAT5B in skeletal muscle between wild-type (WT) and STAT5B^−/−^ mice. Mean (± SEM) concentrations of **a** IGF1 in plasma and **b** *Igf1* mRNA and **c** protein, **d** *Stat5a* mRNA and **e** protein, and **f** *Stat5a* mRNA and **g** protein in quadriceps muscles of male WT (black bars), male STAT5B^−/−^ (light grey bars), female WT (dark grey bars), and female STAT5B^−/−^ (white bars) mice at 6, 12, and 24 weeks of age (*n* = 8 per group). Values for each group are expressed relative to those in male WT mice at 6 weeks of age. Unlike letters within each graph denote significant differences (*P* < 0.05) between groups at each age only. Asterisks denote significant differences (*P < 0.05, ***P < 0.001) at each age only

Unlike concentrations of IGF1 in plasma, concentrations of *Igf1* mRNA and protein in skeletal muscles of wild-type mice were greater in males than in females at all ages and decreased with advancing age in males only (*P* < 0.001; Fig. 4a–c). Concentrations of IGF1 in blood and skeletal muscles of STAT5B^−/−^ mice were reduced in both sexes at 6 weeks of age and in males at all time points (*P* < 0.05 versus wild-type littermates). In contrast, there were no differences in concentrations of IGF1 in blood and skeletal muscles of female STAT5B^−/−^ mice and their female wild-type littermates from 12 weeks of age. As a result, the sexually dimorphic and age-related decrease in the expression of IGF1 in skeletal muscle was abolished in STAT5B^−/−^ mice. Given that STAT5B appears to regulate the production of IGF1 in skeletal muscle throughout the lifespan in male mice and until puberty in female mice, we next sought to determine whether differences in expression of IGF1 in wild-type mice could be due to differences in the expression of STAT5B. Despite no differences in the expression of *Stat5b* mRNA, the approximately twofold greater expression of *Igf1* mRNA in male mice at 6 weeks of age than in female mice of the same age, or than in male mice at 24 weeks of age, was associated with a twofold greater abundance of STAT5B protein (*P* < 0.001; Fig. 4f–g). Conversely, there was no change with advancing age in the abundance of STAT5B protein in skeletal muscles of females or in the abundance of STAT5A protein in either sex (Fig. 4e, g). Concentrations of *Stat5b* mRNA and the abundance of STAT5B protein in skeletal muscles of STAT5B^−/−^ mice were undetectable as expected, while concentrations of *Stat5a* mRNA and the abundance of STAT5A protein in STAT5B^−/−^ mice were reduced at all time points (*P* < 0.05 versus wild-type littermates; Fig. 4d–g).

### Loss of STAT5B reduces the sexually dimorphic expression of SOCS2, CIS, AR, ERα, and myostatin in skeletal muscle

**Fig. 5:**
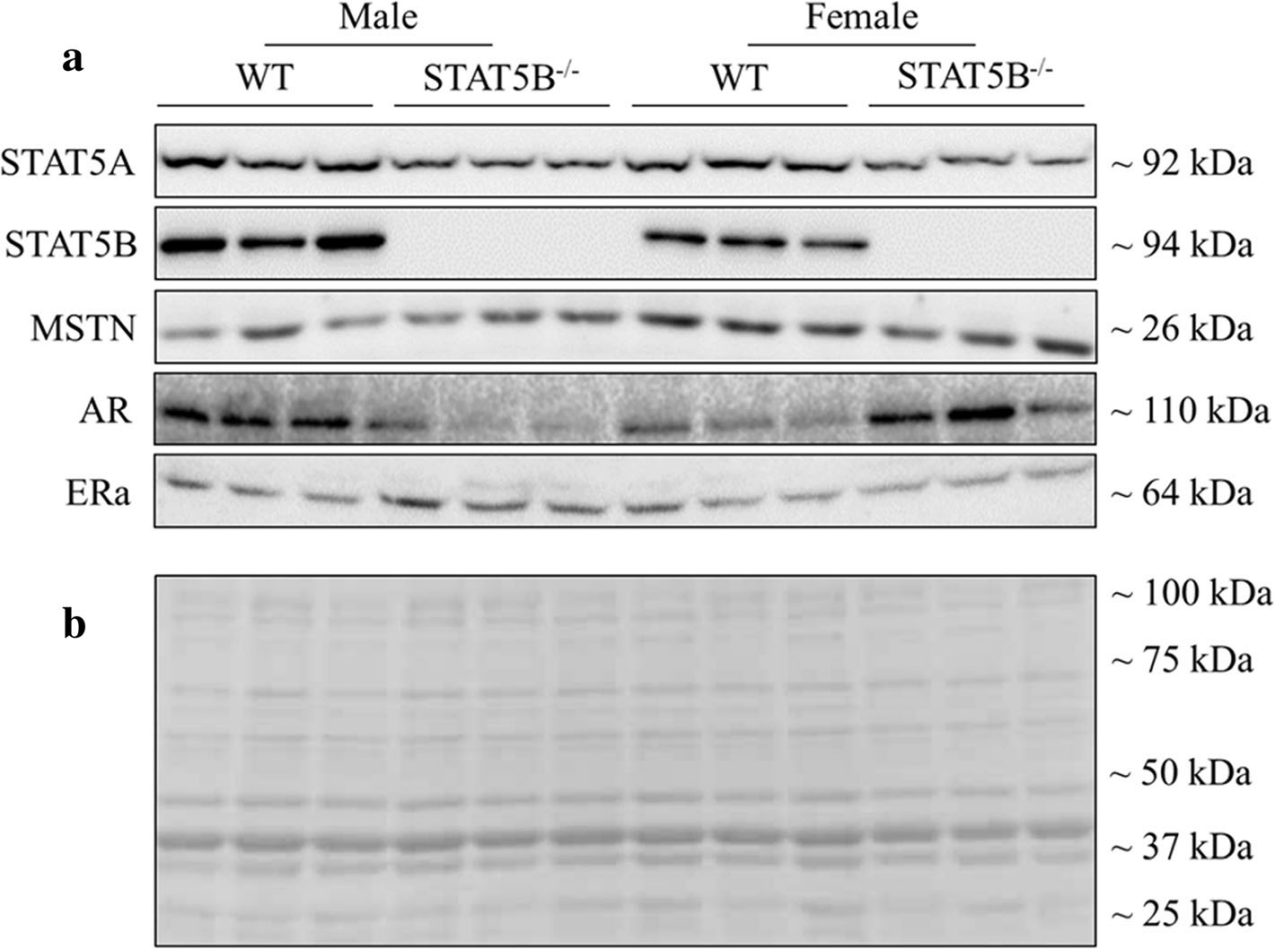
Representative Western blots and Ponceau S stain of proteins from quadriceps muscle of wild-type (WT) and STAT5B^−/−^ mice. **a** Representative Western blots of STAT5A, STAT5B, MSTN, AR, and ERa proteins in quadriceps muscles of male and female WT and STAT5B^−/−^ mice at 6 weeks of age (*n* = 8 per group). **b** The abundance of protein was normalised to the total density of proteins in each lane following staining with Ponceau S

**Fig. 6:**
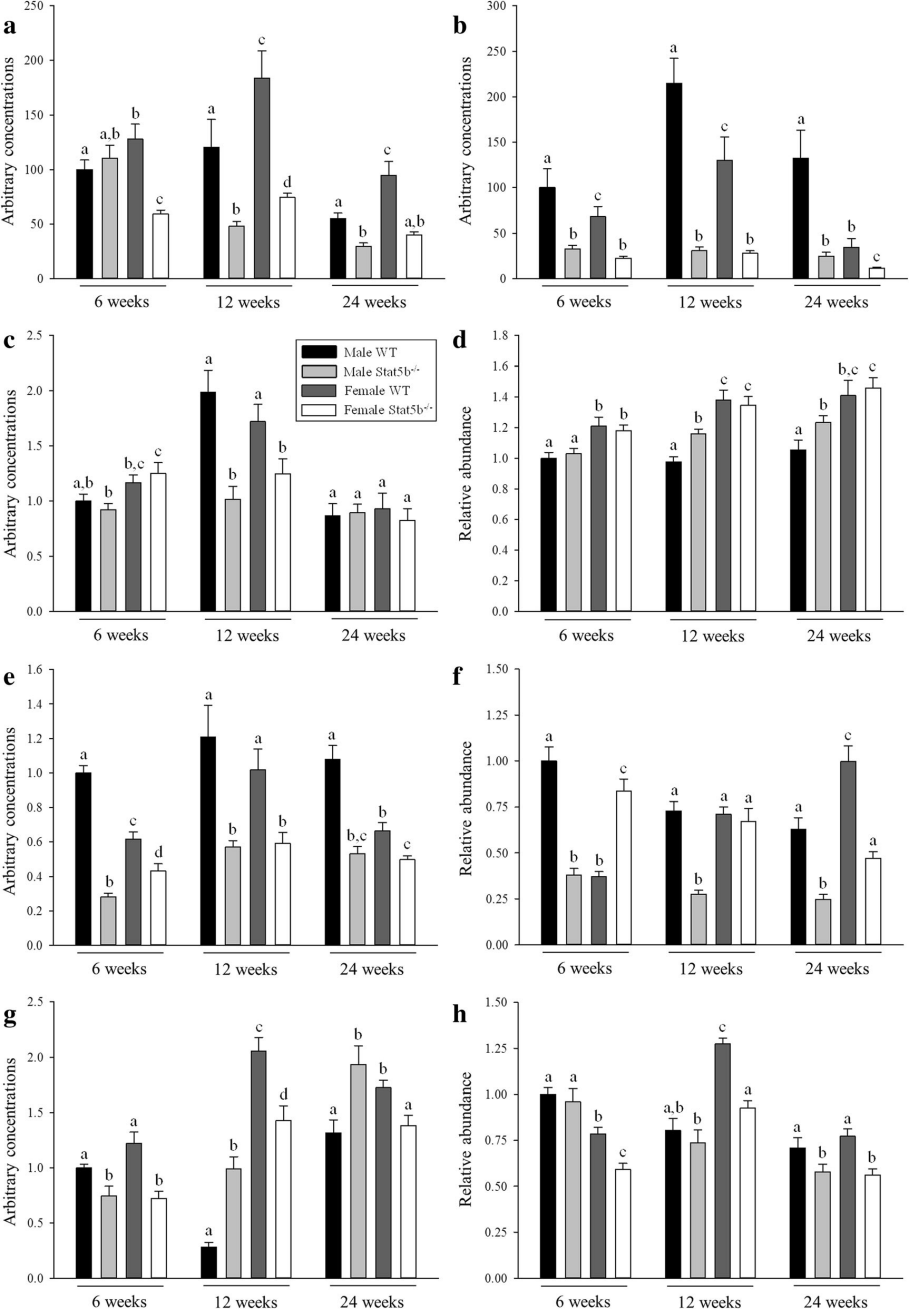
Differences in the expression of SOCS2, CIS, myostatin (MSTN), AR, and ERα between wild-type (WT) and STAT5B^−/−^ mice. Mean (± SEM) expression of **a** *Socs2* mRNA, **b** *Cis* mRNA, **c** *Mstn* mRNA and **d** protein, **e** *Ar* mRNA and **f** protein, and **g** *Era* mRNA and **h** protein in quadriceps muscles of male WT (black bars), male STAT5B^−/−^ (light grey bars), female WT (dark grey bars), and female STAT5B^−/−^ (white bars) mice at 6, 12, and 24 weeks of age (*n* = 8 per group). Values for each group are presented relative to expression in male WT mice at 6 weeks of age. Unlike letters within each graph denote significant differences (*P* < 0.05) between groups at each age only

The expression of inhibitors of STAT5B signalling was sexually dimorphic in skeletal muscles of wild-type mice, with greater expression of *Socs2* mRNA in females and *Cis* mRNA in males (*P* < 0.05; Fig. 6a, b). Concentrations of *Socs2* and *Cis* mRNA were reduced in both sexes of STAT5B^−/−^ mice from 12 weeks of age (*P* < 0.05 versus wild-type littermates), resulting in their sexually dimorphic expression either being reduced (*P* < 0.05) or abolished. Despite no clear effect on the expression of *Mstn* mRNA, loss of STAT5B abolished the sexually dimorphic expression of mature MSTN protein that was evident in wild-type mice from 12 weeks of age (Figs. 5 and 6c, d). Loss of STAT5B also abolished the sexually dimorphic expression of *Ar* and *Erα* mRNA in skeletal muscle by decreasing concentrations of *Ar* mRNA in both sexes and concentrations of *Erα* mRNA in female mice only (*P* < 0.001 versus wild-type littermates; Fig. 6e, g). Accordingly, concentrations of AR and ERα protein were reduced in male and female STAT5B^−/−^ mice (*P* < 0.001 versus wild-type littermates), respectively, with inconsistent changes in the other sex (Fig. 6f, h).

## Discussion

We have shown that when accounting for changes in body composition, STAT5B likely regulates the growth of the skeleton and skeletal muscles in both male and female adult mice. As per previous reports, we found that the body mass of female STAT5B^−/−^ mice is not reduced, but demonstrate that this is due to increased visceral and subcutaneous adiposity despite the decreased skeletal size [7, 8]. Similarly, our data supports previous findings that the absolute mass of the gastrocnemius muscles is not decreased in female STAT5B^−/−^ mice, but we demonstrate that the reduced growth of skeletal muscles in female STAT5B^−/−^ mice is muscle-group specific [9]. The reduction in circulating concentrations of IGF1 and growth of skeletal muscle and the axial and appendicular skeletons of both sexes of STAT5B^−/−^ mice at the onset of puberty demonstrates that STAT5B^−/−^ mice have a similar phenotype to GHR^−/−^, STAT5A^−/−^/STAT5B^−/−^_,_ and STAT5M^−/−^ mice [4, 6, 7]. These findings also address another important paradox, by confirming that the role of STAT5B is more similar between mice and humans than previously thought [13].

**Fig. 7:**
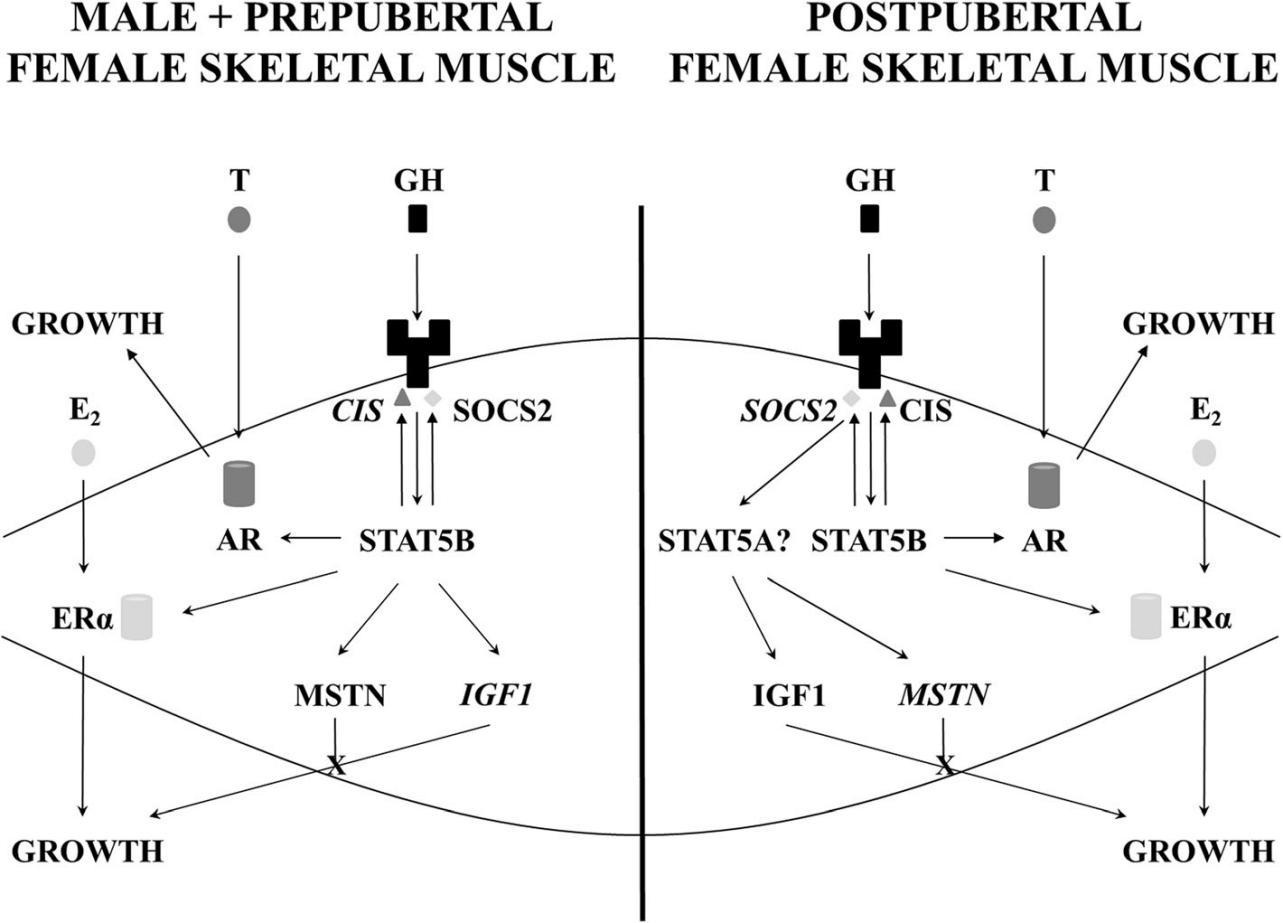
Proposed differences in signalling pathways regulating skeletal muscle growth between and prepubertal female mice and male mice of all ages (left) and adult female mice (right). STAT5B regulates the expression of SOCS2, CIS, AR, and ERα in both sexes and IGF1 and MSTN in female mice before puberty and in male mice of all ages. At puberty, there is a switch in the regulation of IGF1 and MSTN in female mice, and we propose that this is from STAT5B to STAT5A. We have identified that the sexually dimorphic growth of skeletal muscle is due to the onset of sexually dimorphic expression of components of the GH/IGF1 axis at puberty, with greater expression of IGF1 and CIS in males and MSTN and SOCS2 in females (italicised). Actions of GH, testosterone (T), and 17β-estradiol (E_2_) were not assessed in this study

Our findings suggest that STAT5B has sex-specific roles after the onset of puberty. Unlike in male STAT5B^−/−^ mice, the reduced growth of the axial skeleton and the majority of hindlimb muscles, the decrease in concentrations of IGF1 in blood and skeletal muscle, and the correlation between the expression of IGF1 and STAT5B in skeletal muscles of female STAT5B^−/−^ mice had resolved by 12 weeks of age. Therefore, STAT5B appears to regulate the actions of GH throughout the lifespan in male mice, but only until puberty in female mice (Fig. 7). This sex-specific role of STAT5B may be due to the development of sexually dimorphic GH secretion at puberty, with pulsatile secretion of GH, i.e. ‘male pattern’ GH, leading to the formation of STAT5B homodimers, while continuous or ‘female pattern’ secretion of GH results in the formation of STAT5A homodimers and STAT5A/STAT5B heterodimers [31]. These sexually dimorphic differences in dimer formation have been attributed to STAT5A having a longer refractory period to reactivation by GH than STAT5B [32, 33], but we show that these differences may be due to GH signalling in the skeletal muscle being predominately inhibited by SOCS2 in female mice and by CIS in male mice. While CIS internalises the GHR without degradation [34], SOCS2 is a ubiquitin ligase and destroys the GHR [35]. Thereby, SOCS2 likely inhibits GH/STAT5B signalling more than CIS, which is supported by the growth of mice being increased by deletion of SOCS2, but not by deletion of CIS [22, 23]. This sexually dimorphic expression of SOCS2 and CIS likely explains why sexually dimorphic growth is reduced when SOCS2 is deleted or CIS is overexpressed [22, 23]. Moreover, this sexually dimorphic expression of SOCS2 and CIS appears to be tissue-specific because the expression of both *Socs2* and *Cis* mRNA is greater in the livers of female than in male rats [36]. Therefore, the sexually dimorphic patterns of secretion of GH may have different effects in peripheral tissues than the liver.

Our data are consistent with previous studies, wherein STAT5B was reported to regulate the sexually dimorphic expression of *Igf1* and *Ar* mRNA and post-translational processing of myostatin protein in skeletal muscle [9, 15, 20, 37]. Our data are also consistent with a role for STAT5B in regulating the sexually dimorphic expression of *Erα* mRNA in skeletal muscle, which is important because transgenic models have shown that IGF1, myostatin, ERα, and AR all have individual roles in the sexually dimorphic growth of skeletal muscle [3, 15, 18, 19, 38]. In support, others have reported that STAT5A and STAT5B have differential roles in regulating *Erα* and may bind to the 0/B promoter of the gene in rats (C promoter in humans) [39, 40]. Furthermore, the reduced expression of AR and ERα and greater abundance of myostatin protein also likely contribute to the reduced skeletal muscle mass and increased adiposity of STAT5B^−/−^ mice [18, 19, 38, 41]. The loss of androgen signalling may explain why only the growth of the TA and EDL muscles was reduced in female STAT5B^−/−^ mice, because these muscles are fast-twitch muscles that are more androgen responsive than other muscle groups [24, 42]. Reduced activity of androgens may also be why, in contrast to GH-deficient rats, that STAT5B^−/−^ mice have an oxidative rather than a glycolytic shift in the composition of myofibres [43]. Indeed, further studies are required to delineate what effects from loss of STAT5B are due to loss of activity of the GH/IGF1 axis and/or androgens, and whether these effects are muscle group or species specific, particularly given the individual function and rate of growth of each muscle group [2, 44] and the differential expression of AR and ERα in skeletal muscle [45, 46].

Nevertheless, despite a likely switch in the role of STAT5B in regulating IGF1 and myostatin in female skeletal muscle following puberty, STAT5B appears to regulate the expression of AR, ERα, SOCS2, and CIS throughout the lifespan in both sexes (Fig. 7). The loss of STAT5B does not appear to be compensated for by the increased signalling of STAT5A because the expression of *Stat5a* mRNA and protein was reduced. Moreover, the finding that the expression of *Stat5a* mRNA is not reduced in the liver of the same model of STAT5B^−/−^ mouse suggests that like STAT5B, STAT5A has tissue-specific roles [8]. Indeed, we postulate that STAT5A is the principal regulator of the GH/IGF1 axis in adult female skeletal muscle, given that muscle growth is more retarded in female STAT5M^−/−^ and STAT5A^−/−^/STAT5B^−/−^ mice than in female STAT5B^−/−^ mice [6, 7]. Further work is required to characterise the role of STAT5A in skeletal muscle, including identifying whether STAT5A or STAT5B is the most abundant isoform in skeletal muscle, as unlike in the liver, this is currently unknown [47].

Future studies are also required to determine whether the abundance of STAT5B protein reduces with advancing age in tissues other than skeletal muscle in male mice and whether similar changes occur in humans. These age-related changes in STAT5B protein in males and a switch in STAT5B signalling in females would be advantageous in maximising growth while young and reducing the risk of malignancy in adulthood [48, 49]. However, the mechanism for these changes is unclear given that we found that the expression of *Stat5b* mRNA in skeletal muscle does not change with advancing age in either sex.

## Conclusions

We show that STAT5B appears to mediate the anabolic actions of GH in male mice of all ages, but only in female mice until puberty. STAT5B also appears to mediate the actions of androgens and oestrogens in murine skeletal muscle in both sexes, but sexually dimorphic growth persists in STAT5B^−/−^ mice. Demonstrating that the expression of SOCS2 and CIS in skeletal muscle is sexually dimorphic provides new insights into how sexually dimorphic growth and expression of IGF1 and myostatin develop at the onset of puberty.

## Additional files

Additional file 1: **Table S1.** Age and litter size of wild-type (WT) and STAT5B^−/−^ mice. (DOCX 17 kb)

Additional file 2: **Table S2.** Primers used for qPCR. (DOCX 17 kb)

Additional file 3: **Table S3.** Antibodies used in Western blotting. (DOCX 16 kb)

## Data Availability

The datasets used and/or analysed during this study are available from the corresponding author on reasonable request.

## References

[1] Komi PV, Karlsson J (1978). Skeletal muscle fibre types, enzyme activities and physical performance in young males and females. Acta Physiol Scand.

[2] White RB, Bierinx AS, Gnocchi VF, Zammit PS (2010). Dynamics of muscle fibre growth during postnatal mouse development. BMC Dev Biol.

[3] Lupu F, Terwilliger JD, Lee K, Segre GV, Efstratiadis A (2001). Roles of growth hormone and insulin-like growth factor 1 in mouse postnatal growth. Dev Biol.

[4] List EO, Sackmann-Sala L, Berryman DE, Funk K, Kelder B, Gosney ES (2011). Endocrine parameters and phenotypes of the growth hormone receptor gene disrupted (GHR−/−) mouse. Endocr Rev.

[5] Brooks AJ, Waters MJ (2010). The growth hormone receptor: mechanism of activation and clinical implications. Nat Rev Endocrinol.

[6] Klover P, Hennighausen L (2007). Postnatal body growth is dependent on the transcription factors signal transducers and activators of transcription 5a/b in muscle: a role for autocrine/paracrine insulin-like growth factor I. Endocrinology.

[7] Teglund S, McKay C, Schuetz E, van Deursen JM, Stravopodis D, Wang D (1998). Stat5a and Stat5b proteins have essential and nonessential, or redundant, roles in cytokine responses. Cell.

[8] Udy GB, Towers RP, Snell RG, Wilkins RJ, Park SH, Ram PA (1997). Requirement of STAT5b for sexual dimorphism of body growth rates and liver gene expression. Proc Natl Acad Sci U S A.

[9] Oldham JM, Osepchook CC, Jeanplong F, Falconer SJ, Matthews KG, Conaglen JV (2009). The decrease in mature myostatin protein in male skeletal muscle is developmentally regulated by growth hormone. J Physiol.

[10] Morissette MR, Cook SA, Buranasombati C, Rosenberg MA, Rosenzweig A (2009). Myostatin inhibits IGF-I-induced myotube hypertrophy through Akt. Am J Physiol Cell Physiol.

[11] Amirouche A, Durieux AC, Banzet S, Koulmann N, Bonnefoy R, Mouret C (2009). Down-regulation of Akt/mammalian target of rapamycin signaling pathway in response to myostatin overexpression in skeletal muscle. Endocrinology.

[12] Hennebry A, Oldham J, Shavlakadze T, Grounds MD, Sheard P, Fiorotto ML (2017). IGF1 stimulates greater muscle hypertrophy in the absence of myostatin in male mice. J Endocrinol.

[13] Hwa Vivian (2016). STAT5B deficiency: Impacts on human growth and immunity. Growth Hormone & IGF Research.

[14] Yakar S, Liu JL, Stannard B, Butler A, Accili D, Sauer B (1999). Normal growth and development in the absence of hepatic insulin-like growth factor I. Proc Natl Acad Sci U S A.

[15] Paul RGWK, Falconer SJ, Oldham JM, Jeanplong F, Matthews KG, Smith HK, McMahon CD (2018). Transgenic expression of IGF1 and absence of myostatin do not overcome sexual dimorphism of body and muscle size in mice. Submission.

[16] Axell AM, MacLean HE, Plant DR, Harcourt LJ, Davis JA, Jimenez M (2006). Continuous testosterone administration prevents skeletal muscle atrophy and enhances resistance to fatigue in orchidectomized male mice. Am J Physiol Endocrinol Metab.

[17] McCormick KM, Burns KL, Piccone CM, Gosselin LE, Brazeau GA (2004). Effects of ovariectomy and estrogen on skeletal muscle function in growing rats. J Muscle Res Cell Motil.

[18] MacLean HE, Chiu WS, Notini AJ, Axell AM, Davey RA, McManus JF (2008). Impaired skeletal muscle development and function in male, but not female, genomic androgen receptor knockout mice. FASEB J.

[19] Brown M, Ning J, Ferreira JA, Bogener JL, Lubahn DB (2009). Estrogen receptor-alpha and -beta and aromatase knockout effects on lower limb muscle mass and contractile function in female mice. Am J Physiol Endocrinol Metab.

[20] Klover P, Chen W, Zhu BM, Hennighausen L (2009). Skeletal muscle growth and fiber composition in mice are regulated through the transcription factors STAT5a/b: linking growth hormone to the androgen receptor. FASEB J.

[21] Feldman M, Ruan W, Tappin I, Wieczorek R, Kleinberg DL (1999). The effect of GH on estrogen receptor expression in the rat mammary gland. J Endocrinol.

[22] Matsumoto A, Seki Y, Kubo M, Ohtsuka S, Suzuki A, Hayashi I (1999). Suppression of STAT5 functions in liver, mammary glands, and T cells in cytokine-inducible SH2-containing protein 1 transgenic mice. Mol Cell Biol.

[23] Greenhalgh CJ, Bertolino P, Asa SL, Metcalf D, Corbin JE, Adams TE (2002). Growth enhancement in suppressor of cytokine signaling 2 (SOCS-2)-deficient mice is dependent on signal transducer and activator of transcription 5b (STAT5b). Mol Endocrinol.

[24] Augusto V, Padovani CR, Campos GR (2004). Skeletal muscle fiber types in C57BL6J mice. Braz J Morphol Sci.

[25] Soffe Z, Radley-Crabb HG, McMahon C, Grounds MD, Shavlakadze T (2016). Effects of loaded voluntary wheel exercise on performance and muscle hypertrophy in young and old male C57Bl/6J mice. Scand J Med Sci Sports.

[26] White Z, White RB, McMahon C, Grounds MD, Shavlakadze T (2016). High mTORC1 signaling is maintained, while protein degradation pathways are perturbed in old murine skeletal muscles in the fasted state. Int J Biochem Cell Biol.

[27] Lundby C, Nordsborg N, Kusuhara K, Kristensen KM, Neufer PD, Pilegaard H (2005). Gene expression in human skeletal muscle: alternative normalization method and effect of repeated biopsies. Eur J Appl Physiol.

[28] Eaton SL, Roche SL, Llavero Hurtado M, Oldknow KJ, Farquharson C, Gillingwater TH (2013). Total protein analysis as a reliable loading control for quantitative fluorescent Western blotting. PLoS One.

[29] Smith HK, Matthews KG, Oldham JM, Jeanplong F, Falconer SJ, Bass JJ (2014). Translational signalling, atrogenic and myogenic gene expression during unloading and reloading of skeletal muscle in myostatin-deficient mice. PLoS One.

[30] Nuytens K, Tuand K, Fu Q, Stijnen P, Pruniau V, Meulemans S (2014). The dwarf phenotype in GH240B mice, haploinsufficient for the autism candidate gene Neurobeachin, is caused by ectopic expression of recombinant human growth hormone. PLoS One.

[31] Ram PA, Park SH, Choi HK, Waxman DJ (1996). Growth hormone activation of Stat 1, Stat 3, and Stat 5 in rat liver. Differential kinetics of hormone desensitization and growth hormone stimulation of both tyrosine phosphorylation and serine/threonine phosphorylation. J Biol Chem.

[32] Gebert CA, Park SH, Waxman DJ (1999). Down-regulation of liver JAK2-STAT5b signaling by the female plasma pattern of continuous growth hormone stimulation. Mol Endocrinol.

[33] Wang D, Moriggl R, Stravopodis D, Carpino N, Marine JC, Teglund S (2000). A small amphipathic alpha-helical region is required for transcriptional activities and proteasome-dependent turnover of the tyrosine-phosphorylated Stat5. EMBO J.

[34] Uyttendaele I, Lemmens I, Verhee A, De Smet AS, Vandekerckhove J, Lavens D (2007). Mammalian protein-protein interaction trap (MAPPIT) analysis of STAT5, CIS, and SOCS2 interactions with the growth hormone receptor. Mol Endocrinol.

[35] Vesterlund M, Zadjali F, Persson T, Nielsen ML, Kessler BM, Norstedt G (2011). The SOCS2 ubiquitin ligase complex regulates growth hormone receptor levels. PLoS One.

[36] Thangavel C, Shapiro BH (2007). A molecular basis for the sexually dimorphic response to growth hormone. Endocrinology.

[37] Davey HW, McLachlan MJ, Wilkins RJ, Hilton DJ, Adams TE (1999). STAT5b mediates the GH-induced expression of SOCS-2 and SOCS-3 mRNA in the liver. Mol Cell Endocrinol.

[38] Reisz-Porszasz S, Bhasin S, Artaza JN, Shen R, Sinha-Hikim I, Hogue A (2003). Lower skeletal muscle mass in male transgenic mice with muscle-specific overexpression of myostatin. Am J Physiol Endocrinol Metab.

[39] Wilson ME, Westberry JM, Prewitt AK (2008). Dynamic regulation of estrogen receptor-alpha gene expression in the brain: a role for promoter methylation?. Front Neuroendocrinol.

[40] Frasor J, Park K, Byers M, Telleria C, Kitamura T, Yu-Lee LY (2001). Differential roles for signal transducers and activators of transcription 5a and 5b in PRL stimulation of ERalpha and ERbeta transcription. Mol Endocrinol.

[41] Heine PA, Taylor JA, Iwamoto GA, Lubahn DB, Cooke PS (2000). Increased adipose tissue in male and female estrogen receptor-alpha knockout mice. Proc Natl Acad Sci U S A.

[42] Chambon C, Duteil D, Vignaud A, Ferry A, Messaddeq N, Malivindi R (2010). Myocytic androgen receptor controls the strength but not the mass of limb muscles. Proc Natl Acad Sci U S A.

[43] Daugaard JR, Laustsen JL, Hansen BS, Richter EA (1998). Growth hormone induces muscle fibre type transformation in growth hormone-deficient rats. Acta Physiol Scand.

[44] Sheard PW, Anderson RD (2012). Age-related loss of muscle fibres is highly variable amongst mouse skeletal muscles. Biogerontology.

[45] De Naeyer H, Lamon S, Russell AP, Everaert I, De Spaey A, Vanheel B (2014). Androgenic and estrogenic regulation of Atrogin-1, MuRF1 and myostatin expression in different muscle types of male mice. Eur J Appl Physiol.

[46] Baltgalvis KA, Greising SM, Warren GL, Lowe DA (2010). Estrogen regulates estrogen receptors and antioxidant gene expression in mouse skeletal muscle. PLoS One.

[47] Park SH, Liu X, Hennighausen L, Davey HW, Waxman DJ (1999). Distinctive roles of STAT5a and STAT5b in sexual dimorphism of hepatic P450 gene expression. Impact of STAT5a gene disruption. J Biol Chem.

[48] Bartke A, Sun LY, Longo V (2013). Somatotropic signaling: trade-offs between growth, reproductive development, and longevity. Physiol Rev.

[49] Mitra A, Ross JA, Rodriguez G, Nagy ZS, Wilson HL, Kirken RA (2012). Signal transducer and activator of transcription 5b (Stat5b) serine 193 is a novel cytokine-induced phospho-regulatory site that is constitutively activated in primary hematopoietic malignancies. J Biol Chem.

