# Supplementary material for: Regulation of murine skeletal muscle growth by STAT5B is age- and sex-specific
Source: Skelet Muscle. 2019 Jun 24;9:19. doi: 10.1186/s13395-019-0204-3 (PMC6589877; doi:10.1186/s13395-019-0204-3)
Supplement: Supplementary file 1 — Table S1. Age and litter size of wild-type (WT) and STAT5B−/− mice. (DOCX 17 kb) [file 13395_2019_204_MOESM1_ESM.docx]

| **Mice** | | | **Age (weeks)** | **P** | **Litter size** | **P** |
| --- | --- | --- | --- | --- | --- | --- |
| 6 weeks | Male | WT | 5.98 +/- 0.06 | NS | 9.00 +/- 0.46 | NS |
|  |  | Stat5b^-/-^ | 5.98 +/- 0.02 |  | 8.75 +/- 0.77 |  |
|  | Female | WT | 5.98 +/- 0.06 |  | 9.13 +/- 0.48 |  |
|  |  | Stat5b^-/-^ | 5.98 +/- 0.02 |  | 8.88 +/- 0.81 |  |
| 12 weeks | Male | WT | 11.98 +/- 0.05 | NS | 7.38 +/- 0.68 | NS |
|  |  | Stat5b^-/-^ | 12.09 +/- 0.09 |  | 8.87 +/- 0.35 |  |
|  | Female | WT | 12.04 +/- 0.09 |  | 7.13 +/- 0.81 |  |
|  |  | Stat5b^-/-^ | 11.95 +/- 0.05 |  | 8.38 +/- 0.46 |  |
| 24 weeks | Male | WT | 23.95 +/- 0.04 | NS | 6.63 +/- 0.71 | NS |
|  |  | Stat5b^-/-^ | 24.05 +/- 0.04 |  | 6.38 +/- 0.53 |  |
|  | Female | WT | 24.02 +/- 0.06 |  | 5.75 +/- 0.70 |  |
|  |  | Stat5b^-/-^ | 23.98 +/- 0.05 |  | 6.75 +/- 0.49 |  |
