# Supplementary material for: Regulation of murine skeletal muscle growth by STAT5B is age- and sex-specific
Source: Skelet Muscle. 2019 Jun 24;9:19. doi: 10.1186/s13395-019-0204-3 (PMC6589877; doi:10.1186/s13395-019-0204-3)
Supplement: Supplementary file 2 — Table S2. Primers used for qPCR. (DOCX 17 kb) [file 13395_2019_204_MOESM2_ESM.docx]

| **Gene** | **S/AS** | **Sequence (5’-3’)** | **Amplicon size (bp)** |
| --- | --- | --- | --- |
| *Igf1* | S | GCTTGCTCACCTTTACCAGC | 301 |
|  | AS | AAATGTACTTCCTTCTGGGTCT |  |
| *Socs2* | S | ATATCCGTTAAGACGTCAGCTGG | 82 |
|  | AS | TATGATAGAAATCCAATCTGAATTTCCC |  |
| *Cis* | S | ACAGAAGATGCCGGAGGGTACATTC | 152 |
|  | AS | TTGACAAGCAGTTAGAGTCCAGCCG |  |
| *Ar* | S | TACCAGCTCACCAAGCTCCT | 174 |
|  | AS | GATGGGCTTGACTTTCCCAG |  |
| *Era* | S | ATGATTGGTCTCGTCTGGCGCT | 458 |
|  | AS | AGCAGGTCATAGAGGGGCACAACG |  |
| *Mstn* | S | ACCCATGAAAGACGGTACAAG | 288 |
|  | AS | TCATCACAGTCAAGCCCAAAG |  |
| *Stat5a* | S | GAAACCTCTGGAATCTGAAGCC | 159 |
|  | AS | CTGCTTTCGCAAGTACAGGAGT |  |
| *Stat5b* | S | GAGAATTTGCCAGGACGGAA | 51 |
|  | AS | CACGCCATCAAACCACTGC |  |
