# Supplementary material for: Regulation of murine skeletal muscle growth by STAT5B is age- and sex-specific
Source: Skelet Muscle. 2019 Jun 24;9:19. doi: 10.1186/s13395-019-0204-3 (PMC6589877; doi:10.1186/s13395-019-0204-3)
Supplement: Supplementary file 3 — Table S3. Antibodies used in Western blotting. (DOCX 16 kb) [file 13395_2019_204_MOESM3_ESM.docx]

| **Protein target** | **Manufacturer (catalogue number)** | **Species raised in** | **Dilution** |
| --- | --- | --- | --- |
| STAT5A (L-20) | Santa Cruz Biotechnology (sc-1081) | Rabbit, polyclonal | 1:3000 |
| STAT5B (G2) | Santa Cruz Biotechnology (sc-1656) | Mouse, monoclonal | 1:3000 |
| MSTN (H109) | Santa Cruz Biotechnology (sc-28910) | Rabbit, polyclonal | 1:1000 |
| AR (N-20) | Santa Cruz Biotechnology (sc-816) | Rabbit, polyclonal | 1:1000 |
| ERα | Abcam (ab80922-250) | Rabbit, polyclonal | 1:1000 |
